# Transcriptomic and metabolic changes in *Trichoderma reesei* caused by mutation in xylanase regulator 1 (*xyr1*)

**DOI:** 10.1186/s13068-024-02556-8

**Published:** 2024-07-19

**Authors:** Emmi Sveholm, Hans Mattila, Nina Aro, Mari Valkonen, Tanja Paasela, Tiina M. Pakula

**Affiliations:** VTT Technical Research Centre of Finland Ltd, P.O. Box 1000, 02044 Espoo, Finland

**Keywords:** *Trichodermareesei*, XYR1 mutation, Transcriptomics, Metabolomics, Bioreactor

## Abstract

**Background:**

*Trichoderma reesei* is known for its ability to produce large amounts of extracellular proteins and is one of the most important industrially used filamentous fungus. Xylanase regulator 1 (XYR1) is the master regulator responsible for the activation of cellulase and hemicellulase gene expression under inducing conditions. It has been reported that strains with point mutations in certain areas of *xyr1* bypass the need for inducing carbon source, allowing high (hemi)cellulase production even in the presence of glucose. These mutations also change the profile of produced proteins, shifting it more towards xylanase production, and increase the overall protein production in inducing conditions. However, how these mutations alter the metabolism and other cellular processes to cause these changes remains unclear.

**Results:**

In this study, we aimed to explore changes caused by a point mutation in *xyr1* on transcriptomic and metabolic level to better understand the reasons behind the increased protein production in both repressing glucose and inducing lactose conditions. As expected, the expression of many carbohydrate-active enzyme (CAZy) genes was increased in the *xyr1* mutant in both conditions. However, their induction was higher under inducing conditions. The *xyr1* mutant strain built more biomass and produced more extracellular proteins during growth on lactose compared to the wild type *xyr1* strain. Genes involved in oxidoreductive D-galactose catabolism pathway were upregulated in the *xyr1* mutant strain, potentially contributing to the more efficient utilization of lactose. In addition to CAZy genes, clustering and enrichment analysis showed over-representation of mitochondria-related Gene Ontology terms in clusters where gene expression was higher in the *xyr1* mutant, indicating that mitochondria play a role in the altered metabolic state associated with the *xyr1* mutation. Metabolomics revealed that free tyrosine was more abundant in the *xyr1* mutant strain in all measured timepoints, whereas multiple fatty acids were less abundant in the mutant strain on glucose.

**Conclusions:**

The results contribute to more in-depth knowledge on *T. reesei* physiology growing under inducing and repressing carbon sources and gives new insights on the function of the master regulator XYR1. The vast data generated serve as a source for new targets for improved protein production.

**Supplementary Information:**

The online version contains supplementary material available at 10.1186/s13068-024-02556-8.

## Background

The utilization of microorganisms as cellular factories plays a crucial role in converting biomass into sustainable alternatives for fossil-based fuels and materials, as well as for ingredients for food industry. *Trichoderma reesei* is one of the most significant industrially used filamentous fungus due to its ability to secrete considerable amounts of both native cellulolytic enzymes as well as heterologous enzymes [1]. High yields are crucial for profitability in industrial protein production, highlighting the need for continual improvement. Genetic engineering of the production hosts is a common strategy for improving yields.

Numerous transcription factors controlling the expression of cellulase and hemicellulase genes have been identified in *T. reesei*. These include the major transcriptional activator of cellulase and hemicellulase genes XYR1, activator of cellulases (ACE) 2 and ACE3, the carbon catabolite repression protein glucose repressor 1 (CRE1) and negative regulator ACE1 [2, 3]. In *T. reesei*, the production of extracellular proteins is, to a large extent, under carbon catabolite repression (CCR): it is repressed in the presence of easily metabolized carbon sources such as glucose and induced by the presence of more complex carbon sources, such as cellulose or lactose. The main regulator mediating CCR is the transcription factor CRE1 [4, 5]. Under repressing conditions, CRE1 binds to specific sites within the promoters of its target genes [4, 6], including the promoter of XYR1 [7], resulting in the repression of the target genes. Disruption of *cre1* results in increased cellulase production and enables cellulase production also under CCR as seen in the hyperproducer strain RUT-C30 [4, 8]. However, for sufficient production levels, additional inducing agents such as cellulose or disaccharides are needed and disrupting *cre1* alone is not enough to fully circumvent the CCR [9, 10].

XYR1 has been identified as the main transcriptional activator for the expression of cellulase and hemicellulase gene expression in the presence of inducing carbon sources. Its deletion abolishes the cellulase gene expression and adversely affects hemicellulase gene expression. Normally, XYR1 expression is CRE1-dependent and regulated by CCR, rather than being induced directly by the carbon source, and full induction of *xyr1* gene expression requires CRE1 [11, 12], since it has been observed that *xyr1* transcript levels increase more slowly and remain lower in *cre1* deletion mutant [11]. No XYR1-binding sites were found in the upstream region of *xyr1*, indicating that autoregulation is not involved [13]. The transcription factor ACE1 negatively affects the transcription of XYR1, and two binding sites for ACE1 have been identified in the upstream region of *xyr1*, along with 10 CRE1-binding sites [13]. A single point mutation in ACE1 has been shown to improve cellulase production under both inducing and repressing conditions [14]. Mach-Aigner et al*.* [13] hypothesized that the activation of XYR1 may require post-translational modifications. This hypothesis is based on the observation that low levels of XYR1 are present also under repressing conditions, most likely in an inactive form as even low levels of XYR1 can mediate induction. In addition, binding of XYR1 to the promoters of endo-1,4-β-xylanase 1 (*xyn1*) and endo-1,4-β-xylanase 2 (*xyn2*) can be observed under repressing conditions [13, 15, 16]. Another important transcriptional activator for cellulase expression, ACE3, was identified from transcriptional profiling data of *T. reesei* [3]. Overexpression of *ace3* improved both cellulase and hemicellulase production, while deletion abolished cellulase production and slightly reduced hemicellulase expression. ACE3 has been shown to interact with XYR1 to initiate cellulase production [17].

It has been reported that mutations in certain regions of *xyr1* allow cells to bypass the need for induction and enable high production of cellulases and hemicellulases even when growing on the repressing carbon source glucose [9, 10, 18, 19]. These single amino acid mutations, often located in the nuclear receptor-like domain [20], also change the profile of produced proteins, shifting it more towards xylanase production, and increase the overall production of extracellular proteins in inducing conditions. Mello-de-Sousa et al. [20] investigated the structural and functional changes of XYR1 mutant (A824V) protein and observed that the mutation led to a significant loss of helical content in the secondary structure. They also observed reduced binding of XYR1 to the upstream regulatory region of *xyn1*, despite higher *xyn1* expression in the mutant in both repressing and inducing conditions. Two other XYR1 mutations V821F and A873Y are also thought to induce a shift in the same α-helix and lead to similar conformational changes [18]. Further insights from the study by Mello-de-Sousa et al. [20] revealed notable conformational changes induced by carbohydrates in the XYR1 protein. In the wild-type strain, these changes differed between inducing and repressing conditions. However, in the mutant strain, the conformational changes remained consistent irrespective of the carbon source.

In this study, we investigated the transcriptomic and metabolic-level changes caused by a mutation in the major transcriptional activator of cellulase and hemicellulase genes, XYR1, which is known to increase the production of extracellular proteins and facilitate protein production on glucose. We compared *xyr1* mutant strain to wild type *xyr1* strain during growth on lactose and after changing the carbon source from inducing lactose to repressing glucose. Since it has been shown in previous studies that the changes in response to XYR1 mutation cannot be explained merely by altered DNA-binding properties [20], our objective was to gain a deeper understanding of the transcriptional and metabolic changes occurring in the *xyr1* mutant strain that could elucidate the mechanisms behind the increased extracellular protein production. Discovering additional regulatory mechanisms that can be used to improve protein production in industrial *T. reesei* strains are highly valued.

## Results

### Construct design for studying the effects of *xyr1* mutation

**Fig. 1:**
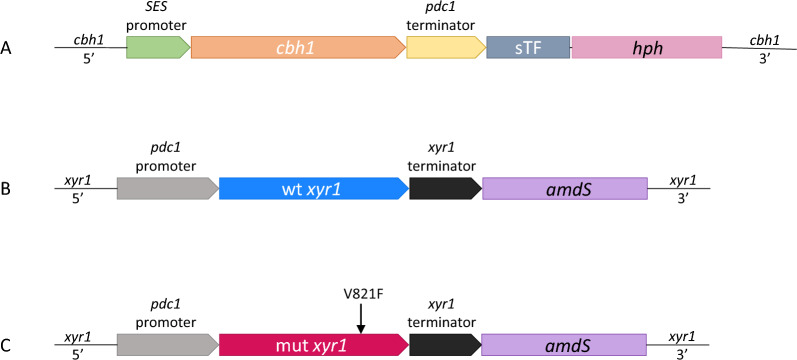
Design of the strains. **A** SESp-cbh1 construct in cbh1 locus, which was the same for both wtXYR1 and mutXYR1. **B** xyr1 locus in wtXYR1. **C** xyr1 locus in mutXYR1

The aim of this study was to investigate the impact of mutated *xyr1* on the gene expression and specific metabolites of *Trichoderma reesei* under inducing and repressing conditions. To achieve this, two genetically modified *T. reesei* QM9414 strains were designed: one expressing wild type *xyr1* (designated as wtXYR1, Fig. 1B) and the other expressing mutated *xyr1* (designated as mutXYR1, which has mutation V821F [10], Fig. 1C). Both coding sequences were overexpressed under constitutive pyruvate decarboxylase (*pdc1*) promoter and replaced the endogenous *xyr1* promoter and coding sequence in order to ensure *xyr1* expression in the presence of glucose, as there is almost no *xyr1* expression under native promoter on glucose in a QM9414 strain with functional CRE1. Since QM9414 produces extracellular proteins only at a very low level under repressing conditions, and we aimed to compare protein production also when the wild type *xyr1* strain is grown on glucose, cellobiohydrolase 1 (*cbh1*) was expressed as a reporter under a synthetic promoter SESp [21, 22], replacing endogenous *cbh1* in its locus (Fig. 1A). Thus, by expressing *cbh1* constitutively, we ensured that protein production can be seen in all conditions and differences in production of extracellular proteins would be easier to detect. The SES system consists of a constitutive synthetic transcription factor (sTF) and an sTF-dependent promoter (SESp), enabling a strong expression of the target gene irrespective of the carbon source [21].

### Bioreactor cultivations in inducing and repressing culture conditions

**Fig. 2:**
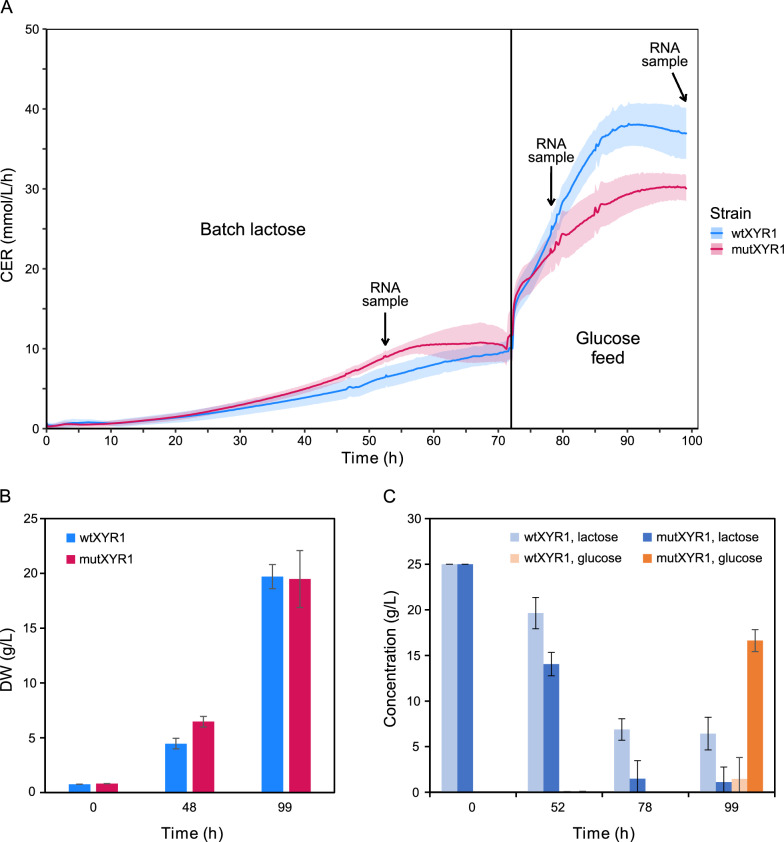
Carbon dioxide evolution rate (CER), cell biomass and lactose and glucose concentrations during bioreactor cultivations. **A** CER during the cultivation. The lines show average CER in the four replicate cultures of both strains and the shade around the lines shows the standard deviation (*n* = 4). The black line at 72 h separates the batch and feed phases of the cultivation and the arrows show approximate RNA sampling times. Lactose was used as carbon source in the batch phase and glucose was used in the feed phase. **B** Biomass during the bioreactor cultivation. Difference at 48 h is statistically significant (*p*-value 0.001). **C** Lactose and glucose concentrations in the supernatants of wtXYR1 and mutXYR1 measured by HPLC during bioreactor cultivations

Precultures were carried out in shake flasks and after 72 h of incubation the biomass and residual lactose were measured. Biomass values were similar for both strains, 3.74 g/L and 3.46 g/L for mutXYR1 and wtXYR1, respectively. Residual lactose was 5.67 g/L and 4.6 g/L for mutXYR1 and wtXYR1, respectively, confirming that the strains did not suffer from carbon source starvation during precultivation. Ambr^®^ bioreactor cultivations started as a batch lactose cultivation, which acted as inducer of extracellular protein production (Fig. 2A). The first RNA samples were taken at 52 h of cultivation, when the carbon dioxide evolution rate (CER) data showed that the cultivations were in the active growth phase and there was enough biomass. Feeding with glucose solution was initiated at around 72 h after starting the cultivation when CER started decreasing and batch lactose was almost consumed according to HPLC analysis. Glucose and lactose concentrations were monitored using HPLC throughout the cultivation (Fig. 2C). Lactose concentration declined until the glucose feeding was initiated and decreased faster in the mutXYR1 cultivations compared to the wtXYR1 cultivations. Feeding with glucose was initiated to switch from cellulase production inducing conditions towards CCR and biomass buildup. This was done to understand why the mutation in *xyr1* enables protein production even under repressing conditions. To ensure glucose repression, the biomass normalized feed rate was set 2 to 2.5 times higher than normally. Glucose accumulation was seen in the mutXYR1 cultivations in the last timepoint during glucose feeding. RNA samples were collected from two timepoints during the glucose feed: at 78 h, 5 h after the start of feeding when CER had stabilized, and in the end of the cultivation at 99 h (Fig. 2A).

During the lactose batch phase, mutXYR1 consumed lactose faster, built slightly more biomass and had higher CER compared to wtXYR1 (Fig. 2B). After the glucose feed was initiated, the CER of wtXYR1 rose higher than that of mutXYR1, resulting in similar end cell biomass at 99 h between mutXYR1 and wtXYR1 strains (Fig. 2A).

The amount of extracellular protein was measured using Bradford protein assay. The amount of protein produced by mutXYR1 strain was higher than that of the wtXYR1 in all three timepoints (Figure S1B). In both strains, the major cellulase gene *cbh1* was expressed under the strong synthetic SESp promoter and its gene expression was significantly higher than the expression of any other gene. Therefore, a substantial portion of the detected protein was CBH1, forming a baseline for extracellular protein expression and reflecting the general capacity of the strains to produce extracellular proteins. At all three timepoints, the difference in total extracellular protein concentration between the strains was statistically significant (*p*-value < 0.01). However, the difference occurred during batch lactose phase and the protein production rates were similar between the last two timepoints during glucose feeding. At the end of the cultivation, mutXYR1 had produced approximately 2.3 g/L proteins and wtXYR1 1.7 g/L. The composition of produced proteins was also analyzed with SDS-PAGE (Figure S1A). It showed distinct differences in the profile of produced proteins.

### Transcriptome-level changes in response to *xyr1* mutation and carbon source

To analyze the differences in gene expression between the strains expressing either wild type XYR1 or mutated XYR1, samples of the bioreactor cultivations were collected during the batch cultivation phase on lactose as well as at two timepoints during the glucose feeding phase and submitted for RNA sequencing. Samples were collected from four replicate cultivations per strain. Overall alignment rates per sample against *T. reesei* version 2.0 genome [23] were between 81.8% and 93.9%, the average being 87.8%. The presence of the V821F mutation [10] was confirmed by aligning reads against *T. reesei* v2.0 genome using Integrative genomics viewer IGV [24]. Both *xyr1* mutant and wild type *xyr1* were expressed in all three timepoints due to overexpression under *pdc1* promoter. The expression level of *xyr1* mutant was slightly lower than the expression of wild type *xyr1* during batch lactose phase and late glucose feed. Differential gene expression analysis was carried out with DESeq2 [25]. Genes with adjusted *p*-value < 0.05 and log_2_ fold change > 1 or < −1 were considered to be differentially expressed. List of differentially expressed genes is presented in Table S1.

**Fig. 3:**
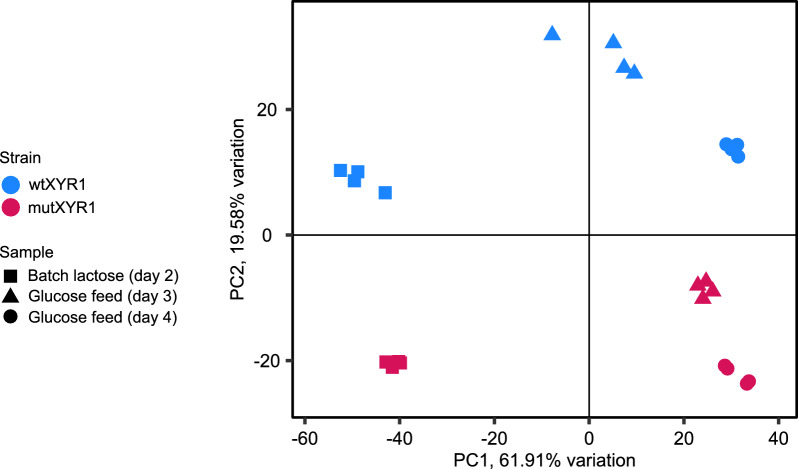
Principal component analysis (PCA) of the RNA-sequencing samples. Data points of wtXYR1 and mutXYR1 are marked with blue and red, respectively. Batch lactose samples are represented by squares, glucose feed samples collected on day three are marked with triangles and glucose samples collected on day four are marked with circles

A Pearson correlation analysis and a principal component analysis (PCA) were conducted to evaluate the reliability of the sequencing data and the replicate samples (Fig. 3). PCA of the dataset containing all three timepoints from mutXYR1 and wtXYR1 strains yielded multiple principal components, of which the three first ones together explain 88% of the variation. Replicate samples clustered close together in the PCA plot and had high Pearson correlations (r > 0.98). PCA suggests that the main source of variation (PC1, 61.91%) is the carbon source. Samples collected from batch lactose phase cluster on the opposite side of the plot than the two sample sets collected from the glucose feed phase. The highest dispersion between strains regarding the carbon source occurred following the initiation of glucose feeding on day 3. In the PCA plot, this can be seen when the mutXYR1 samples from day 3 cluster closely with the samples collected on day 4, while wtXYR1 samples from day 3 cluster more in the center of the plot, indicating that the transition to glucose utilization was slower in the wild-type samples. The second most significant source of variation seems to be the strain (PC2, 19.58%). MutXYR1 samples cluster towards the negative end of the axis corresponding to PC2, while wtXYR1 samples are closer to the positive end of the same axis in the PCA plot.

It is well known that the expression of *T. reesei* genes is greatly affected by the carbon source available to the fungus, especially when comparing the gene expression during cultivation on inducing and repressing conditions [26]. This was clearly visible in the PCA and can also be visualized in the expression heatmap of all *T. reesei* genes (Fig. 3, Figure S2). The heatmap shows averaged, scaled, and centered expression values of each gene in each strain and condition. A black line within the heatmap separates the batch lactose and glucose feed samples. In the batch lactose samples, genes at the top of the heatmap exhibit higher expression levels compared to the average, while genes at the bottom show lower expression levels. Conversely, in the two glucose feed samples, gene expression behaves oppositely, with more highly expressed genes located at the bottom of the heatmap. The heatmap not only illustrates the influence of the carbon source, but also highlights differences between strains under the same cultivation conditions (Figure S2).

### Exploring enriched functional groups through clustering-based analysis

Different expression patterns present in the data were investigated by employing soft clustering for all known expressed genes in *T. reesei* using Mfuzz package [27]. Genes were clustered into 50 clusters in order to separate different functions into different clusters. To elucidate the processes or functions associated with each cluster, over-representation analysis was conducted independently for each cluster using clusterProfiler [28, 29]. This analysis utilized both Gene Ontology (GO) [30, 31] terms and Kyoto Encyclopedia of Genes and Genomes (KEGG) [32] pathways. GO annotations for *T. reesei* were made using Blast2GO [33]. Enriched GO terms and KEGG pathways for each cluster are listed in Additional file 1. Multiple clusters were identified in which GO terms associated with CAZymes and mitochondria were prominent and were chosen for more in-depth characterization. On the other hand, some clusters were showing very different expression profiles between the mutant and wild-type strains, but the clusters did not have any over-represented terms. All cluster profiles are presented in Figure S4.

**Fig. 4:**
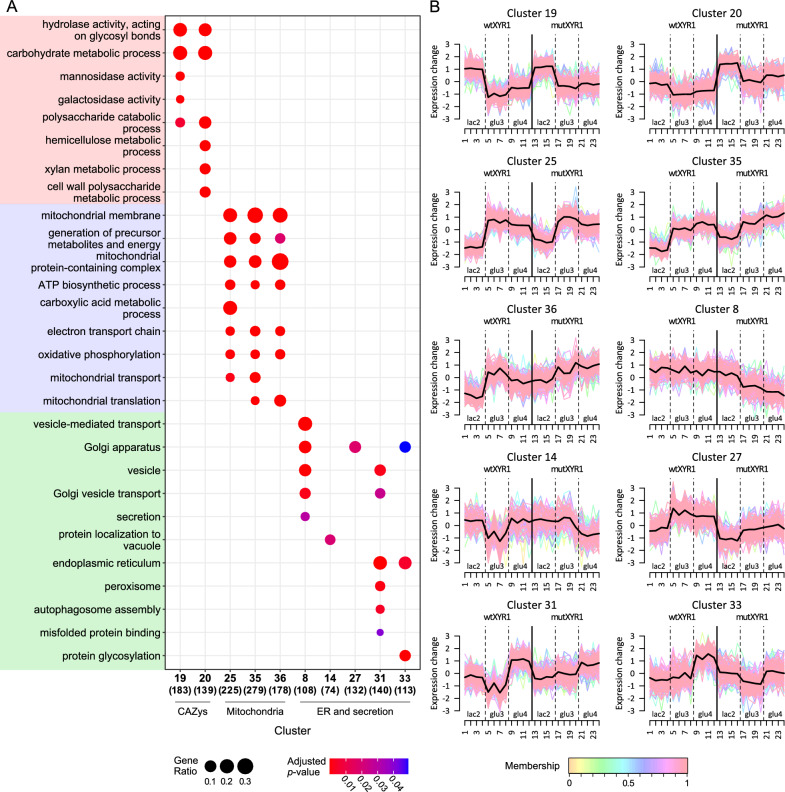
Over-represented GO terms in selected clusters and expression profiles of the clusters. **A** Dotplot of selected over-represented GO terms in clusters related to CAZymes, mitochondria and ER and secretion. The size of the dot corresponds to gene ratio, i.e., the number of genes belonging to the GO term in question in the cluster compared to all genes belonging to that GO term and the number in parenthesis under each cluster number is the number of identified genes in the cluster. **B** Gene expression profiles of the selected clusters. Samples 1–12 correspond to wtXYR1 strain and samples 13–24 correspond to mutXYR1 strain. Biological replicates are shown separately, and the dotted lines separate the timepoints from each other. The horizontal black line in the middle is the cluster center and the colored lines represent the expression profiles of individual genes. The color of the line indicates the membership value. The higher the membership value, the better the gene fits in the cluster

The function of endoplasmic reticulum (ER) and the secretory pathway in general is important for efficient protein production. Therefore, clusters over-represented with ER- and secretion-related terms were also chosen for more detailed analysis to see if any differences can be detected between the strains. Identified clusters, cluster profiles and selected terms related to the chosen functions are represented in Fig. 4.

CAZy genes mainly fall into two separate clusters (clusters 19 and 20) (Fig. 4), but individual CAZy genes can also be found scattered in other clusters. In general, CAZy genes are expressed on a higher level on lactose compared to glucose feed in both strains. CAZy genes in cluster 19 have a similar expression in both strains on lactose and a slightly higher expression in the mutant on glucose. Genes in cluster 19 include cellobiohydrolases 2 (*cbh2*), β-glucosidase 1 (*bgl1*) and endo-β-1,4-glucanase 1 (*egl1*). In cluster 20, CAZy genes are expressed on a higher level in the mutant in all three timepoints. This cluster includes CAZy genes with the highest log_2_ fold changes between the strains. Most upregulated CAZy genes in the mutant were endo-β-1,4-xylanase 5 (*xyn5*) on batch lactose and early glucose feed and β-xylosidase 1 (*bxl1*) on late glucose feed. Over-represented GO terms in both clusters include, e.g., “hydrolase activity, acting on glycosyl bonds” and “carbohydrate metabolic process”. There were also over-represented GO terms that were present in one cluster but not in the other. These include “mannosidase activity” and “galactosidase activity” in cluster 19 and “hemicellulose metabolic process”, “xylan metabolic process” and “cell wall polysaccharide metabolic process” in cluster 20. Other clusters that include CAZy genes but did not have CAZy-related over-represented GO terms were for example cluster 17, in which the expression is lower in the mutant on lactose and similar during glucose feed. This group of genes was identified from gene expression heatmap including only differentially expressed CAZy genes according to Häkkinen et al. [34] (Figure S6). CAZy genes such as three candidate β-glucosidases (*cel3b, bgl3j* and *bgl3i)* and α-L-arabinofuranosidase 1 (*abf1*) can be found in this cluster.

Three clusters (25, 35, 36) with enriched mitochondria-related GO terms were also identified (Fig. 4). In general, these clusters were quite similar in profile: expression was lower on lactose and increased during glucose feed. Also, the expression levels were in general higher in the mutXYR1 strain compared to the wtXYR1 strain, the exception being cluster 25, in which the expression levels on glucose feed were similar in both strains. Common over-represented GO terms in all three clusters were cellular component terms related to different mitochondrion parts, such as “mitochondrial membrane” and “mitochondrial protein-containing complex”. Also, terms such as “ATP biosynthetic process”, “electron transport chain” and “oxidative phosphorylation” were among over-represented GO terms in all three clusters. KEGG pathway oxidative phosphorylation was also enriched in all three clusters.

Two clusters that contained enriched ER-related terms (clusters 31 and 33) and three clusters containing secretion-related terms (8, 14, 27) were identified (Fig. 4). In cluster 31, the expression was generally higher during glucose feed compared to batch lactose, with the exception of a drop in expression in the wtXYR1 strain on glucose feed day 3. In cluster 33, the expression levels increase during cultivation in the wtXYR1 strain and stay the same in the mutXYR1 strain, with a drop in expression again on day 3. During batch lactose and glucose feed day 4 in cluster 31, the expression levels of the strains are similar, possibly slightly lower in the mutXYR1 in the glucose feed day 4 timepoint. Expression is higher in the mutXYR1 strain in the glucose feed day 3 timepoint. In cluster 33, expression is slightly higher in the mutXYR1 strain on lactose and lower in both glucose feed timepoints compared to the wtXYR1 strain. Both clusters are enriched with terms related to different parts of ER, such as “endoplasmic reticulum”. Other enriched terms in the clusters differ from each other. Cluster 31 is enriched with terms related to autophagy, peroxisome and vesicles, for example “autophagosome assembly”. Cluster 33 on the other hand is enriched with terms related to glycosylation, such as “protein glycosylation”. Both clusters are over-represented with genes from the KEGG pathway protein processing in endoplasmic reticulum. Enriched KEGG pathways in cluster 31 also include autophagy and phagosome and in cluster 33 N-Glycan biosynthesis.

Clusters associated with secretion exhibited diverse expression profiles, reflecting the different biological processes enriched in the clusters. However, all clusters displayed lower expression in the mutXYR1 strain compared to the wtXYR1 strain in at least one timepoint. In cluster 8, this seemed to be the case especially in the two glucose feed timepoints, whereas in cluster 14, the biggest difference was in the last glucose feed timepoint, where the expression was lower in the mutXYR1 strain compared to wtXYR1 strain. In cluster 27, expression levels seemed to be lower in the mutXYR1 strain throughout the cultivation. Secretion-related clusters also differed more regarding the terms enriched in them. Cluster 8 had multiple terms related to Golgi, such as “Golgi apparatus” and “Golgi vesicle transport”, as well as terms like “vesicle-mediated transport” and “secretion”. Cluster 27 had only one enriched term, “Golgi apparatus”. Cluster 14 on the other hand had multiple enriched vacuole related terms, e.g., “protein localization to vacuole”.

**Fig. 5:**
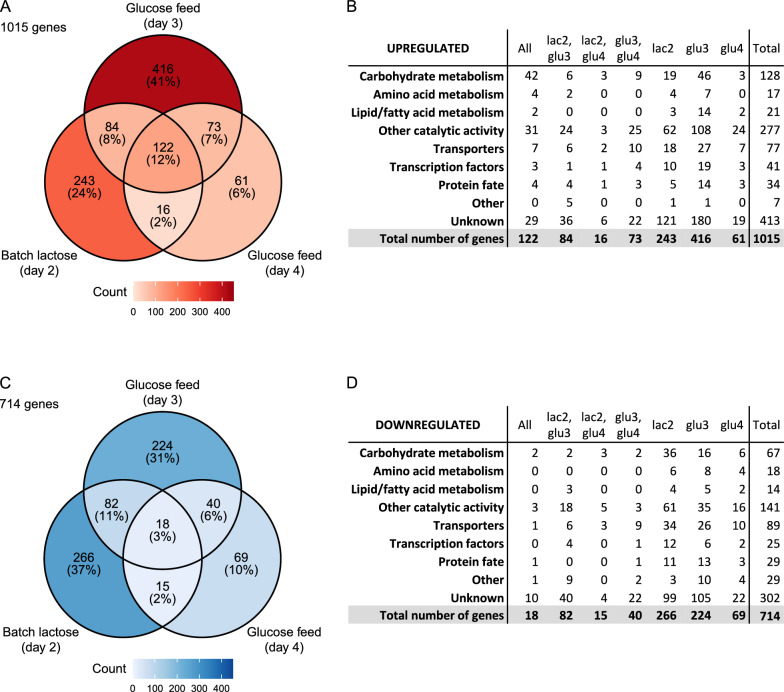
Venn diagrams of up- and down-regulated genes (adjusted *p*-value < 0.05, |log_2_ fold change|> 1) in mutXYR1 strain compared to wtXYR1 strain and the functional groups in which the genes belong to. **A** Upregulated genes. **B** The functional groups of the upregulated genes. **C** Downregulated genes. **D** The functional groups of the downregulated genes

Venn diagrams were drawn and common genes among different samples were investigated (Fig. 5A, C), aiming to elucidate the possible reasons why mutXYR1 strain produces more proteins on lactose and can produce proteins also when growing on repressing carbon source glucose without induction. Over-representation analysis was also conducted for different Venn groups using clusterProfiler [28, 29] and both GO terms and KEGG pathways. Genes in Venn groups were categorized into functional groups based on available annotations, such as GO terms and PANNZER annotations [35], if enough data were available of the genes (Fig. 5B, D). When investigating the number of differentially expressed genes (*p*-value < 0.05, |log_2_ fold change|> 1) in the mutXYR1 strain compared to wtXYR1 strain in the different timepoints, it can be observed that the total number of differentially expressed genes is higher during the batch cultivation phase on lactose than during the last glucose feed timepoint (Fig. 5, Figure S3). However, the highest number of upregulated genes in the mutXYR1 strain was observed in the early glucose feed timepoint, shortly after the initiation of glucose feed.

There were 122 genes that were upregulated in mutXYR1 compared to wtXYR1 and 18 genes that were downregulated in all three conditions (Fig. 5, Table S1). Many of the upregulated genes were CAZys such as xylanases *xyn1-5*, β-xylosidase (*bxl1*) and α-glucuronidase (*glr1*). Genes involved in regulation for example putative kinases and transcription factors, and genes encoding for transporters were also upregulated. Genes linked to carbohydrate metabolism formed the largest group among those upregulated in all conditions, followed by genes categorized as “other catalytic activity” (Fig. 5B). There were also 7 genes annotated as transporters and 3 putative transcription factors. A putative transcription factor annotated as cutinase regulator (TRIRE0072076) exhibited a notable upregulation in the mutXYR1 strain, with approximately 32-fold higher expression compared to the wtXYR1 strain across the three timepoints. This finding was particularly interesting given the absence of orthologues, e.g., in *Aspergillus*, *Neurospora*, or *Saccharomyces* according to EnsemblFungi [36]. Multiple GO terms related to carbohydrate catabolic processes were over-represented in the mutXYR1 strain in all three timepoints and GO term “extracellular region” was enriched among genes that were downregulated in the mutXYR1 strain. Most of the downregulated genes had no predicted function and were annotated as unknowns (Fig. 5D).

There were 73 genes that were upregulated and 40 genes that were downregulated in the mutXYR1 strain in both glucose feed samples but not in batch lactose sample (Fig. 5, Table S1). About a third of the upregulated genes were annotated as unknowns, while another third were categorized as “other catalytic activity” (Fig. 5B). About a half of the downregulated genes in both glucose feed timepoints were annotated as unknowns and a quarter of the genes were annotated as transporters (Fig. 5D). Among the genes in these Venn categories, there are multiple upregulated metabolism-related genes such as putative dehydrogenases and polyketide synthase, putative regulators and transporters. GO term “ABC-type transporter activity” was enriched among genes that were downregulated in the mutXYR1 strain in both glucose timepoints but not on lactose.

There were 243 genes that were upregulated and 266 genes that were downregulated in the mutXYR1 strain only on lactose (Fig. 5, Table S1). A half of the upregulated and more than third of the downregulated genes were categorized as unknowns (Fig. 5BD). Both Venn groups also included multiple putative transporters and transcription factors and genes related to carbohydrate metabolism. Enriched GO terms among the upregulated genes on lactose were, e.g., "heme binding", "tetrapyrrole binding", "monooxygenase activity" and "iron ion binding". GO terms related to “carbohydrate transport”, “carbohydrate derivative catabolic process” and “solute:proton symporter activity” were enriched among the downregulated genes on lactose.

All the genes shown in the Venn diagrams are listed in Table S1.

### Lactose metabolism genes are upregulated in *xyr1* mutant strain

The mutXYR1 strain produced more extracellular proteins and reached a higher dry weight during growth on lactose than the wtXYR1 strain. Therefore, we were interested to find out whether this could be attributed to an increased lactose utilization capacity in the mutXYR1 strain. Lactose metabolism starts with the hydrolysis of lactose to D-glucose and β-D-galactose by β-galactosidases [37]. In *T. reesei*, lactose is thought to be hydrolyzed extracellularly and BGA1 (β-galactosidase 1, TRIRE0080240) is considered the main extracellular β-galactosidase in lactose hydrolysis [38]. It has also been observed that increasing β-galactosidase activity decreases cellulase production on lactose [38]. Intriguingly, we did not observe increased *bga1* expression in the mutXYR1 strain on lactose (Table S4), suggesting that increased protein production in the mutXYR1 strain may not be directly linked to enhanced lactose hydrolysis.

D-Galactose is metabolized by two separate pathways, leading to glycolysis: the Leloir pathway and an alternative, oxidoreductive pathway [37, 39]. While, e.g., *S. cerevisiae* uses solely the Leloir pathway for D-galactose catabolism, *T. reesei* uses also the oxidoreductive pathway during growth on lactose [40]. We found increased expression of all four genes (aldose reductase encoding *xyl1*, galactitol dehydrogenase encoding *lad1*, L-xylo-3-hexulose reductase encoding *lxr4* and D-sorbitol dehydrogenase encoding *xdh1*) in the oxidoreductive D-galactose catabolism pathway in the mutXYR1 strain in all three timepoints, ranging from 1.4-fold increase to 10.6-fold increase in the expression levels (*p*-value < 0.01) (Table S4). Highest increase in expression in the mutXYR1 strain was in *xyl1* (D-xylose reductase 1). Its deletion has previously been linked with decreased growth on lactose, decreased expression of *bga1* and reduced cellulase expression [40]. The expression of putative Leloir pathway genes did not differ as much between the strains, however *T. reesei* has multiple possible homologs to *S. cerevisiae* GAL10.

### Metabolite-level changes caused by *xyr1* mutation

Metabolomics was used to get a better insight in the differences observed between the strains. During the bioreactor cultivations, the mutXYR1 strain was producing more extracellular proteins compared to the wtXYR1 strain, but also growing better on lactose (Fig. 2, Figure S1). The differences in extracellular protein levels were smaller during glucose feed. While RNA-seq is giving an overview of the differences on transcriptional level, metabolomics elucidates how these differences affect the downstream metabolic responses.

Metabolomics was carried out using a gas chromatography–mass spectrometry (GC–MS) based method. Twenty metabolites were analyzed quantitatively, including 13 free amino acids, 4 fatty acids, 2 TCA cycle intermediates and glyceric acid. For these, specific concentrations were obtained. The remaining metabolites were analyzed qualitatively, and relative quantification was employed to obtain an estimation of their abundancy.

**Fig. 6:**
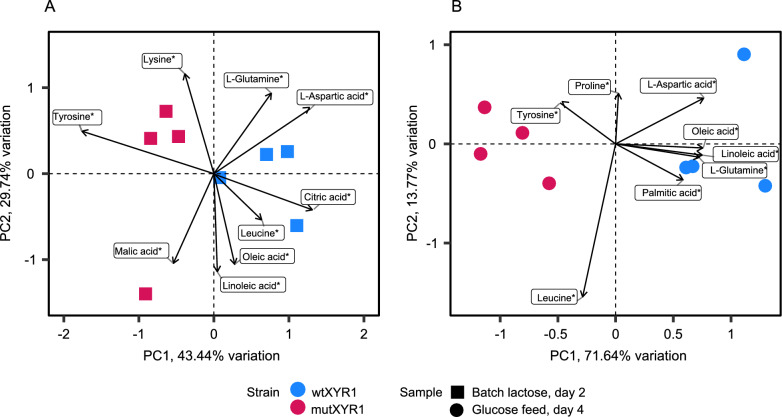
Principal component analysis of the quantified metabolites in **A** batch lactose timepoint from day 2 and **B** glucose feed timepoint from day 4. WtXYR1 samples are represented with blue color and mutXYR1 samples with red color. Batch lactose samples are marked with squares and late glucose feed samples with circles. Arrows represent metabolite loadings, i.e., the direction and weight of how the metabolites affect the principal components

Data from the quantified metabolites were used to perform PCA. In the PCA with all the samples (Figure S5), carbon source seemed to be the biggest source of variation, explaining 51.6% of the variation (PC1). Variation due to differences in strains seems to be associated with PC2, explaining 22.0% of the variation. Given the substantial impact of the carbon source, individual PCAs were performed for batch lactose and the last glucose feed timepoints to separate the strain-specific effect on lactose and glucose (Fig. 6). After removing the variation caused by carbon source, strain seemed to be associated with PC1 and was the biggest source of variation. On batch lactose, PC1 explained 43.4% of the variation whereas on the last glucose feed timepoint, 71.6% of the variation was explained by PC1. Therefore, it seems that when it comes to metabolite abundancies, the strains differed more from each other on glucose than on lactose.

Metabolites, which had the highest contribution to PC1 in the separate PCA plots partly differed on batch lactose and late glucose feed. In both analyses, tyrosine was more abundant in the mutXYR1 strain, with component loadings of −0.59 and −0.25 contributing to PC1 for lactose and glucose, respectively. The difference in metabolite concentration was also statistically significant (*p*-value < 0.01), determined using two-tailed Student’s *t*-test assuming equal variances. Citric acid and L-aspartic acid were more abundant in the wtXYR1 strain both on batch lactose and on late glucose feed. Fatty acids palmitic acid (16:0), oleic acid (18:1) and linoleic acid (18:2) were less abundant in the mutXYR1 strain compared to the wtXYR1 strain during late glucose feed, whereas on batch lactose there was no significant difference between the strains. L-Glutamine was also more abundant in the wtXYR1 strain in the late glucose feed timepoint.

**Fig. 7:**
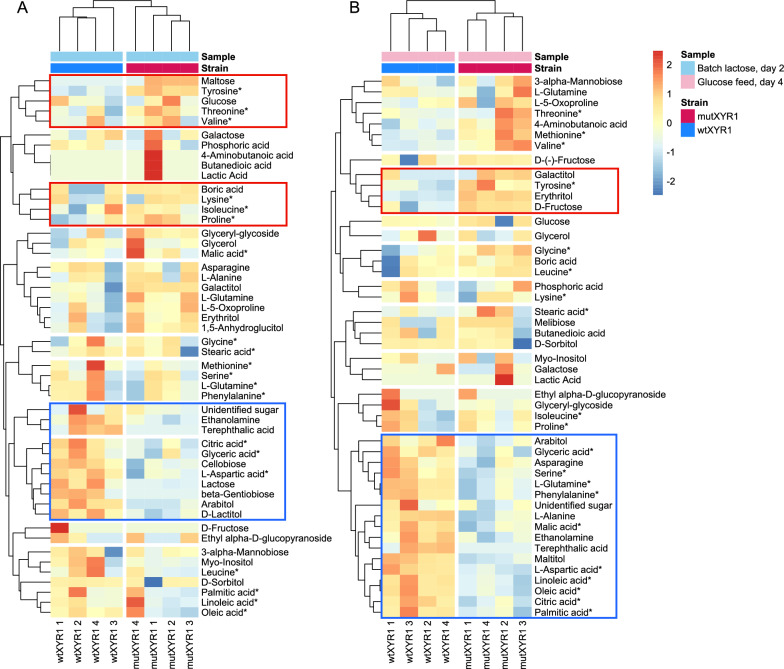
Heatmaps visualizing the differences in metabolomics data obtained from the mutXYR1 strain and the wtXYR1 strain bioreactor cultivations. The colors indicate deviations from the mean metabolite abundance across all samples. Metabolites that appeared more abundant in the mutXYR1 compared to wtXYR1 strain are marked with red boxes, whereas metabolites that appeared less abundant are marked with blue boxes. Metabolites marked with an asterisk were measured quantitatively, the rest were evaluated qualitatively using an internal standard. **A** Metabolite abundancies from batch lactose samples. **B** Metabolite abundancies from the last glucose feed timepoint

Heatmaps were generated to visualize the differences in metabolite abundancies between the strains on batch lactose day 2 and glucose feed day 4 (Fig. 7). This time, data from both quantitatively and qualitatively analyzed metabolites were used. Data were log_2_ transformed and Pareto scaled prior to heatmap construction and heatmaps were generated for each timepoint separately to see the effect of the *xyr1* mutation.

In addition to the quantitatively analyzed fatty acids, L-aspartic acid, citric acid and tyrosine that were already discussed, there were clusters of qualitatively analyzed metabolites in all timepoints in which the metabolite abundancies seemed to differ between the strains. Clusters of metabolites in which the metabolite abundancies appeared higher in the mutant are highlighted with red boxes in the heatmaps. Amino acids such as threonine and proline seemed to be more abundant in the mutXYR1 strain on lactose based on the heatmap, but the difference was not statistically significant. Disaccharide maltose and sugar alcohol galactitol were more abundant (*p*-value < 0.05) on lactose. In the late glucose feed timepoint, for example sugar alcohol erythritol and D-fructose were more abundant in the mutXYR1 strain (*p*-value < 0.01).

Metabolites that appeared less abundant in the mutXYR1 strain based on the heatmaps are marked with blue boxes. For example, arabitol, cellobiose and lactose were less abundant in the mutXYR1 strain on lactose (*p*-value < 0.05). There were more metabolites in the late glucose feed timepoint that were less abundant in the mutXYR1 strain compared to metabolites that seemed more abundant. For example, amino acids such as serine, L-glutamine and L-alanine were less abundant in the mutXYR1 strain (*p*-value < 0.05). Also, malic acid and arabitol were less abundant in the mutXYR1 strain (*p*-value < 0.05).

## Discussion

In this study, we aimed to gain a deeper understanding of the mechanisms behind the increased extracellular protein production and of bypassing the need for induction in the mutXYR1 strain. To achieve this, we employed RNA-sequencing and metabolomics analysis under both inducing and repressing conditions.

During the cultivation, differences in the cell biomass and total protein concentration between the strains were most evident during batch lactose phase. The mutXYR1 strain achieved higher cell biomass and total extracellular protein concentration compared to the wtXYR1 strain at this stage. Although the total protein concentration was higher in the mutXYR1 samples throughout the cultivations, protein production rates during glucose feed were similar in both strains and differences in the amount of cell biomass disappeared (Figure S1B). A substantial amount of the measured total extracellular protein consists of the reporter protein CBH1. This is because, under the strong synthetic promoter, the expression of *cbh1* far exceeds that of any other CAZy genes. It is also possible that the higher biomass achieved by the mutXYR1 strain caused or at least affected the differences seen in the amount of total extracellular proteins between the strains on lactose. An unexpected observation in the mutXYR1 strain cultivations was the glucose accumulation into the supernatant towards the end of the cultivation, suggesting slower glucose intake and consumption rates in the mutXYR1 strain compared to the wtXYR1 strain (Fig. 2C).

RNA-sequencing was conducted to study the transcriptional differences between the mutXYR1 and the wtXYR1 strains. A heatmap was used to visualize the impact of carbon source and the mutation in *xyr1* on gene expression at the whole transcriptome level (Figure S2). Changes can be seen on two levels: changing the carbon source from lactose to glucose had a dramatic effect on the gene expression profile, and differences between the mutXYR1 strain and the wtXYR1 strain are also evident in different timepoints. The heatmap also supports what is known about the gene expression in *T. reesei* under repressing and inducing conditions [26]. Genes encoding cellulolytic enzymes, e.g., *cbh2* and *egl1* and *egl2* have significantly higher expression in the wtXYR1 strain in batch lactose timepoint compared to the two glucose feed time points, indicating that the glucose repression was on as expected.

It has been shown in previous RNA-seq studies that *xyr1* deletion abolishes the expression of multiple CAZy genes [41, 42]. Furthermore, it has been shown that certain point mutations in *xyr1* enhance the expression of cellulases and xylanases [9, 10, 18, 19]. Our data were consistent with these known regulatory functions of XYR1. Multiple CAZy genes were upregulated in response to the mutation in *xyr1*. In addition to these findings, our data highlight the diverse nature of CAZy regulation. Groups of CAZys with varying responses were identified, including a group in which the expression was lower in the mutXYR1 strain on lactose compared to the wtXYR1 strain with no difference between the strains during glucose feed (Figure S6). However, in all groups, the expression was higher on batch lactose than during glucose feed. Therefore, despite the *xyr1* mutation enabling production under repressing conditions, the expression levels were generally lower than in inducing conditions. Notably, genes encoding for proteins related to xylan and hemicellulose catabolic processes had the strongest positive reaction to the *xyr1* mutation, whereas mannosidase and galactosidase activities increased only slightly during glucose feed, indicating an impact of the *xyr1* mutation to these specific enzymatic activities. These findings underscore the selective influence of the *xyr1* mutation on distinct CAZy groups, particularly those involved in xylan and hemicellulose degradation. In these previous RNA-seq studies, multiple transporters, such as ABC and MFS transporters, were observed to be differentially expressed in *xyr1* deletion strain in the presence of cellulose, sophorose and glucose [41, 42]. Similarly, our data show that transporters belonging to different transporter families were differentially expressed in response to the *xyr1* mutation.

It seems that the mutXYR1 strain adapts differently to the transition from one carbon source to another compared to wtXYR1 (Fig. 3). At the time of collecting the early glucose feed samples, the transcriptional profile of the mutXYR1 strain was more similar to the transcriptional profile of the late glucose feed samples and the dispersion between the replicates was small. In contrast, the transcriptional profile of the wtXYR1 samples taken during early glucose feed resembled more that of the batch lactose samples. Venn diagrams support the findings from the PCA, indicating that the most notable differences in gene expression occur at the transition phase following the initiation of glucose feed (Fig. 5). After the glucose feed continued, the number of differentially expressed genes decreased. The number of differentially expressed genes between the mutXYR1 strain and the wtXYR1 strain was much higher on batch lactose compared to late glucose feed.

Interestingly, mitochondria-related functions seemed to be more expressed in the mutXYR1 strain both on batch lactose and glucose feed and were also more expressed during glucose feed compared to lactose. The over-representation of mitochondrial terms in clusters that showed increased gene expression in the mutXYR1 strain remains unclear. Potential explanations include whether the mutXYR1 strain is able to utilize and generate energy more efficiently, facilitating improved protein production or if increased protein production creates a greater demand for energy, subsequently activating mitochondrial gene expression. In previous studies, it has been observed that the physiological state of mitochondria affects cellulase gene expression in *T. reesei* [43, 44]. For instance, decreased oxygen tension and inhibition of electron transport chain downregulated the expression of *cbh1* and *egl1* [43]. This suggests that the observed changes in mitochondrial-related functions in the mutXYR1 strain may indeed play a role in shaping the cellular environment leading to enhanced protein production. However, a comprehensive understanding of these phenomena requires further investigations [43, 44].

We were hypothesizing whether the increased extracellular protein production caused by the mutation in *xyr1* could at least partly be due to differential expression of genes associated with the secretory pathway. The use of CBH1 as a reporter protein allowed us to examine the potential differences in the capacity of the secretory pathway between the two strains. The *cbh1* gene was expressed under a synthetic promoter and was therefore not under XYR1 regulation. Thus, its expression was expected to be very high and similar in both strains, causing a similar load on the secretory pathway. Possible differences in the expression of secretory pathway genes could therefore be caused by the *xyr1* mutation rather than differences in the secretory load. However, there were no clear differences in the expression of genes encoding for various functions of the protein secretion pathway between mutXYR1 and wtXYR1. The genes encoding for functions in the ER were not more expressed in the *xyr1* mutant, therefore differences in protein synthesis do not seem to explain the increased protein production. However, we cannot completely rule out the possibility that differences in the secretory pathway may not be detectable at the transcriptional level.

In this study, it was observed that the mutXYR1 strain produced more extracellular proteins and reached a higher dry weight during growth on lactose than the wtXYR1 strain. Therefore, we were interested to find out whether the mutXYR1 strain was able to utilize lactose more efficiently compared to wtXYR1. Based on the increased expression of oxidoreductive D-galactose catabolism pathway genes (aldose reductase encoding *xyl1*, galactitol dehydrogenase encoding *lad1*, L-xylo-3-hexulose reductase encoding *lxr4* and D-sorbitol dehydrogenase encoding *xdh1*) [39], it seems possible that the mutXYR1 strain could utilize galactose derived from lactose more efficiently than the wtXYR1 strain. It has also been previously observed that deletion of *xyr1* leads to growth impairment and reduced extracellular β-galactosidase activity on lactose [40], which supports the findings suggesting XYR1 involvement in lactose utilization.

We also observed higher galactitol abundance in the mutXYR1 strain compared to the wtXYR1 strain on lactose (approximately 3.8 times higher) but no difference on glucose feed in metabolomics analysis. Galactose can be converted to galactitol during galactose catabolism [39]. These findings suggest that the mutXYR1 strain may exhibit a distinctive metabolic profile compared to wtXYR1, potentially favoring the oxidoreductive D-galactose catabolism pathway during lactose utilization. Further investigations into pathway preference and its implications on protein production could reveal valuable insights into the mechanisms underlying the observed differences between mutXYR1 and wtXYR1 strains.

Metabolomics showed accumulation of free tyrosine in the mutXYR1 strain compared to the wtXYR1 strain in all three timepoints, but we did not see upregulation of the putative tyrosine biosynthesis encoding genes (KEGG pathway tre00400) in the RNA-seq data (Table S2). Tyrosine degradation pathway (KEGG pathway tre00350) from tyrosine to fumarate or acetoacetate did not seem to be downregulated either. The only difference seen in the RNA-seq data was the clear downregulation of the putative tyrosinase encoding genes (TRIRE0045445, TRIRE0050793) in the mutXYR1 strain on lactose. These genes had a log_2_ fold change of −1.6 and −3.2, respectively. Based on the KEGG pathway tre00350, the two tyrosinases seem to have roles in the conversion of tyrosine to L-DOPA and dopaquinone and they could be involved in melanin biosynthesis, however not much is known about melanins in *T. reesei*. Gene TRIRE0045445, named *tyr2* in a previous study, was found to be secreted outside the cell and its overexpression led to a production of a stronger brown color around the colonies [45]. It has been suggested that tyrosinase production can be induced under stress conditions and that tyrosinases are involved in melanin production, which could improve survival [45]. Decreased tyrosinase production and increased free tyrosine levels in the mutXYR1 strain could imply decreased melanin production and altered stress tolerance. We also saw a decrease in the free tyrosine levels in mutXYR1 in late glucose feed compared to batch lactose, which was not seen in wtXYR1 strain, where tyrosine levels remained similar in both timepoints. This would also support the results from RNA-seq data, where the putative tyrosinase genes were strongly downregulated in mutXYR1 compared to wtXYR1 on lactose but the expression levels in both strains were similar in the last glucose feed timepoint. However, we have not studied if the mutXYR1 strain has altered stress tolerance or reduced amount of melanin.

Additionally, a decrease in fatty acid pools in the mutXYR1 strain compared to wtXYR1 was observed in the metabolomics analysis in the last glucose feed timepoint, but no difference was seen on lactose. To study whether this could imply that the storage lipids are consumed by fatty acid β-oxidation and used as energy source for growth and protein production and therefore degradation would be increased in the mutant, the expression of putative genes participating in β-oxidation in *T. reesei* was explored. In a previous study, in silico screen was conducted to identify enzymes in the β-oxidation pathways in *T. reesei* and other fungi and localization prediction indicated that *T. reesei* possesses both peroxisomal and mitochondrial β-oxidation pathways [46]. We did not see clear differences in the identified peroxisomal or mitochondrial β-oxidation genes between the strains. Putative genes participating in β-oxidation in *T. reesei* that were identified by [46] are listed in Table S3. However, fatty acids have multiple other roles in cells, such as in signaling and sporulation and serving as cell membrane constituents [47, 48]. It is possible that differences in the amounts of fatty acids between the strains could also reflect differences in these functions.

RNA-sequencing is a powerful tool to analyze whole transcriptome-level changes that can aid in dissecting pathways that are affected by mutation in XYR1 and cause improved protein production capacity. It has been shown that XYR1 is interacting with other transcriptional regulators such as ACE3 and other proteins such as ATP-dependent chromatin remodelers which then impact the expression of (hemi)cellulase genes [17, 49]. Since it has been shown, counterintuitively, that the mutation in XYR1 results in reduced DNA-binding affinity [20], it would be interesting to study further whether the mutation impairs protein–protein interactions (PPI), thereby causing the observed phenotypes. Unfortunately, large-scale PPI studies involving XYR1 have not been conducted nor is there PPI network data available for *T. reesei*. Furthermore, there is no data available whether XYR1 activity is regulated by post-translational modifications, such as phosphorylation or methylation, as has been shown for instance for ACE1 [14]. These studies could improve understanding of the XYR1 regulatory network, since not all regulatory effects are observable at the transcriptional level, and proteins responsible, e.g., for post-translational modifications can be difficult to identify among hundreds of differentially expressed genes.

## Conclusions

Transcriptional and metabolomic profiles of a *xyr1* mutant strain and a strain with a wild type *xyr1* were compared during a bioreactor cultivation first under inducing conditions and then under repressing conditions. Significant changes were seen on both levels. Mitochondria-related genes were upregulated in the *xyr1* mutant strain compared to the strain with wild type *xyr1*, and the mutation seemed to enable more efficient utilization of lactose through upregulated galactose oxidoreductive pathway. Many genes involved, for instance, in signaling, regulation and transport were differentially expressed in the studied strains and cultivation conditions and can be useful candidates for strain improvement. Increased tyrosine levels and decreased fatty acid levels were also observed in the *xyr1* mutant strain.

This study provides insights into the transcriptional and metabolic dynamics of the *xyr1* mutant strain, shedding light on its enhanced protein production and ability to bypass the need for induction. The results obtained in this study provide a base for further studies particularly in the exploration of specific pathways such as mitochondrial functions and galactose metabolism.

## Methods

### Plasmid and fungal strain construction

*T. reesei* strain QM9414 was obtained from VTT Culture Collection (Espoo, Finland) and used as a host for the genetically engineered strains. Three different plasmids were used to generate the *Trichoderma reesei* strains for this study. The first plasmid was used to target the SESp-*cbh1* construct to replace the endogenous *cbh1* locus in QM9414 strain. The plasmid contained *cbh1* under synthetic SES promoter (SESp [21]), *pdc1* terminator, 1.7–1.9 kb flanks for targeting the expression cassette into *cbh1* locus and an expression cassette encoding the synthetic transcription factor (sTF) [21]). *Hph* encoding for hygromycin B phosphotransferase was used as the selection marker. This generated strain acted as a host for the two other transformations. The second plasmid was used to target the expression cassette with mutated *xyr1* (V821F, [10]) into the endogenous *xyr1* locus. The plasmid contained mutated *xyr1* under *pdc1* promoter and native *xyr1* terminator, acetamidase (amdS) selection and 1.5 kb flanks for targeting the expression cassette into *xyr1* locus. It was transformed into the SESp-*cbh1* strain, replacing the endogenous *xyr1* locus. The third plasmid was otherwise the same as the second plasmid but had the wild type *xyr1* instead of the mutated version. It was also transformed into the SESp-*cbh1* strain, replacing the endogenous *xyr1* locus.

*T. reesei* protoplasts were transformed as described by Penttilä et al. [50]. Digested expression cassettes were treated with T7 exonuclease (New England Biolabs, USA) to generate 3’ single stranded overhangs and protoplasts were treated with *mus53* siRNA (Sigma, USA) to facilitate the integration. Used siRNA sequences were GUCAUCAUCGGCGGCUACU, GGCUUAAGGUAAAGCCCGA and GACUCAUCCUGCCGGACAA. *mus53* is a part of the non-homologous end joining (NHEJ) pathway and its deletion improves homologous recombination and therefore targeted integration of DNA at a specific locus [51]. In acetamide transformations, Triton top agar was added 24 h after the transformation to restrict the growth of colonies.

Transformation plates and top agar consisted of trMM minimal media (15 g/L KH_2_PO_4_, 5 g/L (NH_4_)_2_SO_4_, 1 mL/L trace element solution (5 g/L FeSO_4_⋅7H_2_O, 1.6 g/L MnSO_4_⋅H_2_O, 1.4 g/L ZnSO_4_⋅7H_2_O, 3.7 g/L CoCl_2_⋅6H_2_O)) supplemented with 20 g/L glucose and 182.2 g/L sorbitol to act as an osmotic stabilizer and the appropriate selection. For hygromycin selection, 150 µg/mL hygromycin was used and for acetamide selection, 10 mM final concentration was used. After the transformation, transformants were re-streaked into transformation plates without sorbitol but supplemented with 0.1% (v/v) Triton X-100 to restrict the mycelial growth. For constructing a strain with a capability to produce CBH1 on glucose, triton plates were also supplemented with 125 µg/mL hygromycin for selection. For constructing the *xyr1* mutant strain and its control strain with the wild type *xyr1*, acetamide selection was used and (NH_4_)_2_SO_4_ was left out of the transformation plates and top agar, and acetamide and CsCl_2_ were added after autoclaving to final concentrations of 10 mM and 12.6 mM, respectively. Triton top agar was otherwise the same as top agar, but without sorbitol and supplemented with 0.1% (v/v) Triton X-100.

*T. reesei* transformants were screened for 3’ and 5’ integration with colony PCR using Phire Plant kit (Thermo Fisher Scientific, USA). Transformants that were positive for both integrations were purified into uninuclear clones and screened again to confirm the right integration. The copy number of *xyr1* was confirmed by qPCR. Genomic DNA was extracted from the candidate transformants using a phenol–chloroform extraction and a fragment of the *xyr1* gene was amplified in order to confirm the right mutation or the lack of mutation in the transformants by sequencing. The newly generated strains were named mutXYR1 (QM9414 SESp-*cbh1* pdc1p-*xyr1*mut) and wtXYR1 (QM9414 SESp-*cbh1* pdc1p-*xyr1*wt).

To prepare spore suspensions, *T. reesei* was cultivated on potato dextrose agar (PDA) for 4–7 days until sporulation occurred, the spores were harvested in a buffer containing 0.8% (w/v) NaCl, 20% (v/v) glycerol and 0.025% (v/v) Tween-20 and the suspension was filtered through a cotton filter. Spore suspensions were stored at −80 °C.

### Bioreactor cultivations

The precultures for bioreactor cultivations were cultivated in 2000-mL Erlenmeyer shake flasks using 200 mL of preculture media (2% (w/v) lactose trMM + 0.2% (w/v) peptone) and the final spore concentration of 2 × 10^8^/L of preculture media. Shake flasks were incubated in an orbital shaker in + 28 °C at 200 rpm (orbit diameter 25 mm). Seed cultures were transferred to bioreactors after 72 h of preculture. All parallel cultivations were started from the same pooled seed culture. The volume of transferred inoculum was 10% of the bioreactor’s initial volume of 210 ml. Bioreactor cultivations were carried out with Sartorius Ambr^®^ 250 High Throughput -system (Sartorius, Germany) in four replicate reactors. The batch media contained 30 g/L of lactose, 15 g/L of KH_2_PO_4_, 12 g/L of (NH_4_)_2_SO_4_, 0.59 g/L of MgSO_4_, 2 mL/L of trace element solution and 1.6 mL/L of CaCl_2_. All reactors were fed with 550 g/L glucose solution. Antifoam agent Struktol J637A was added when needed.

### RNA extraction, library construction and sequencing

In total, 24 mycelium samples from bioreactor cultivations were collected for RNA-sequencing from three different timepoints: during the batch lactose phase and at two timepoints during the glucose feeding phase. For each strain and timepoint, four biological replicates were sampled. Samples were filtered through a filter paper, washed with sterile RO water, and immediately frozen in liquid nitrogen, after which they were stored in −80 °C.

Total RNA was extracted from mycelia using TRIzol (Thermo Fisher Scientific, USA) according to manufacturer’s instructions with small modifications. Mycelium samples were ground using mortars under liquid nitrogen into fine powder and about 50 µL of powder was transferred into a 2 mL tube and 1 mL of TRIzol was immediately added. Mixture was vortexed and incubated at room temperature for 5 min, after which it was centrifuged (12000 × g, 4 °C, 10 min) and the supernatant was transferred to a new tube. After this, the protocol was continued according to manufacturer’s instructions. RNA was then purified using RNeasy Mini kit (Qiagen, Germany) according to manufacturer’s instructions, except that 950 µl of 94 vol-% ethanol was added instead of 250 µl. RNA concentration was measured using NanoDrop ND-1000 (NanoDrop Technologies Inc. Wilmington, DE, USA) and the integrity was verified with Agilent 2100 Bioanalyzer (Agilent Technologies, Palo Alto, CA, USA).

Extracted and purified RNA samples were sent for RNA-sequencing in Source BioScience (Cambridge, UK). Stranded rRNA depletion libraries were prepared using Next Ultra II Directional RNA Library Prep kit (New England Biolabs, USA). Sequencing of the rRNA depletion libraries were done using Illumina NovaSeq 6000 to yield approximately 20 million 150 bp paired-end reads per sample.

### RNA-seq data analysis

Reads were quality filtered and trimmed using FastP v0.20.0 [52] and the quality of the raw sequence data was evaluated using FastQC v0.11.8 [53]. Reads were then aligned to *T. reesei* genome version 2.0 [23] with GFF annotations using HISAT2 v2.1.0 [54]. Sam-files were sorted and compressed with SAMtools v1.9 [55] and the alignments were assembled and quantified using StringTie v2.0.4 [56]. Downstream analyses were carried out in R v4.2.2 [57], using both CRAN and Bioconductor [58] packages. Quality of the replicates was assessed with principal component analysis (PCA) using package PCAtools v2.10.0 [59]. Differential gene expression analysis was carried out with R package DESeq2 v1.34.0 [25], considering genes with adjusted *p*-value < 0.05 and log_2_ fold change > 1 or < -1 to be differentially expressed. Venn diagrams were drawn in R using ggVennDiagram v1.2.3 [60]. Soft clustering of the genes was done with R package Mfuzz v2.58.0 [27]. For enrichment analysis, functional GO annotations were generated for *Trichoderma reesei* v2.0 protein sequences using OmicsBox Blast2GO suite [33] according to the basic pipeline and using fungal sequences as reference in BLAST. Over-representation analysis was done in R using clusterProfiler v4.6.2 enricher function [28, 29], GO.db v3.16.0 [61] to retrieve GO term names and rrvgo v1.10.0 [62] to find corresponding parent terms for GO terms using the closest supported organism *Saccharomyces cerevisiae*. The dataset supporting the conclusions of this article is available in NCBI's Gene Expression Omnibus and is accessible through GEO Series accession number GSE260567 (https://www.ncbi.nlm.nih.gov/geo/query/acc.cgi?acc=GSE260567).

### Metabolomics

Mycelium samples were first freeze-dried overnight using Alpha 2-4 LSCBasic freeze-dryer (Christ, Germany). Metabolomics analyses were carried out based on a protocol by Fiehn [63] with modifications, specified as follows. In the sample preparation and clean-up, mixture of acetonitrile, isopropanol and water was degassed by sonication. After adding 5 mg of dried mycelium sample in step 13, two zirconium balls were added, and the sample was vortexed and shook as in the protocol. In step 19, supernatant was removed to an autosampler vial. During derivatization, in step 4, 25 µL of methoxyamine hydrochloride (MeOX) was used instead of 10 µl. In step 5, derivatization was carried out for 1 h at 45 °C. In step 7, 90 µL of N-methyl-N-(trimethylsilyl)-trifluoroacetamide (MSTFA) + 10 µL retention index (RI) mixture was added. After that, derivatization was carried out for 0.5 h at 45 °C. Then sample was transferred to an insert and 25 µl of hexane was added.

For the GC–MS analysis, samples (injection volume 1 µL) were analyzed on an Agilent 7890A gas chromatograph (GC) coupled to an Agilent 5975C mass selective detector (MSD). A split ratio 1:10 was used in the injection and the injector and MSD interface temperatures were 260 °C and 230 °C, respectively. GC was equipped with a DB5-MS column (30 m, ID 250 µm, film thickness 0.25 µm; Agilent 122–5532) and helium was used as a carrier gas. The column was operated with a temperature program from 40 °C (2 min) to 160 °C (5 °C/min), from 160 °C to 325 °C (30 °C/min, hold time 18.5 min). The mass spectra were recorded over a 35–600 amu at 2.6 scans/s.

Raw data were processed with Agilent MassHunter MS Quantitative Analysis (targeted) and with MassHunter Unknowns Analysis (non-targeted) software. Data were Pareto scaled [64], PCA was done in R using package PCAtools v2.10.0 [59] and heatmaps were generated with R packages pheatmap v1.0.12 [65] and dendextend v1.17.1 [66], with centered and scaled data. Statistic testing was done using two-tailed Student’s *t*-test assuming equal variances.

### Supplementary Information

Additional file 1.

Additional file 2.

Additional file 3.

## Data Availability

The sequence dataset supporting the conclusions of this article is available in NCBI's Gene Expression Omnibus and is accessible through GEO Series accession number GSE260567 (https://www.ncbi.nlm.nih.gov/geo/query/acc.cgi?acc=GSE260567).
