# Supplementary material for: Transcriptomic and metabolic changes in Trichoderma reesei caused by mutation in xylanase regulator 1 (xyr1)
Source: Biotechnol Biofuels Bioprod. 2024 Jul 19;17:106. doi: 10.1186/s13068-024-02556-8 (PMC11265206; doi:10.1186/s13068-024-02556-8)
Supplement: Supplementary file 2 — Additional file 2. [file 13068_2024_2556_MOESM2_ESM.docx]

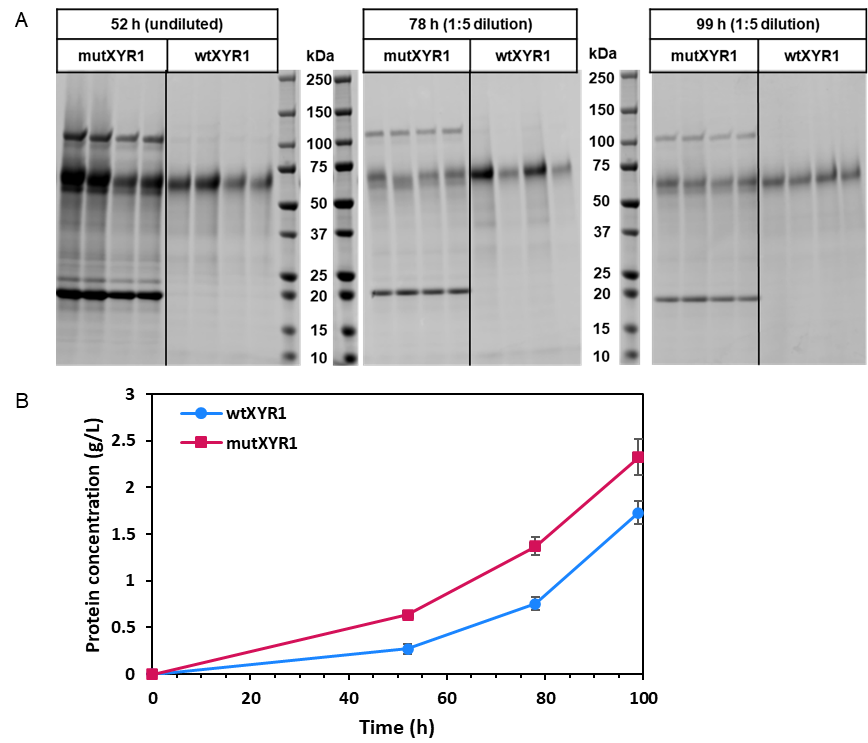


Figure S1. Total extracellular protein production during bioreactor cultivations. A) SDS-PAGE gels of supernatants collected from RNA sampling timepoints of wtXYR1 and mutXYR1 bioreactor cultivations. Samples collected at 52 h was not diluted and samples collected at 78 h and 99 h were diluted 1:5. B) Total extracellular protein concentrations measured from bioreactor culture supernatants of wtXYR1 and mutXYR1 from the same timepoints. In both strains, CBH1 reporter band can be seen around 70 kDa. In addition, bands around 20 kDa and 100 kDa are visible in mutXYR1 samples but not in wtXYR1 samples.


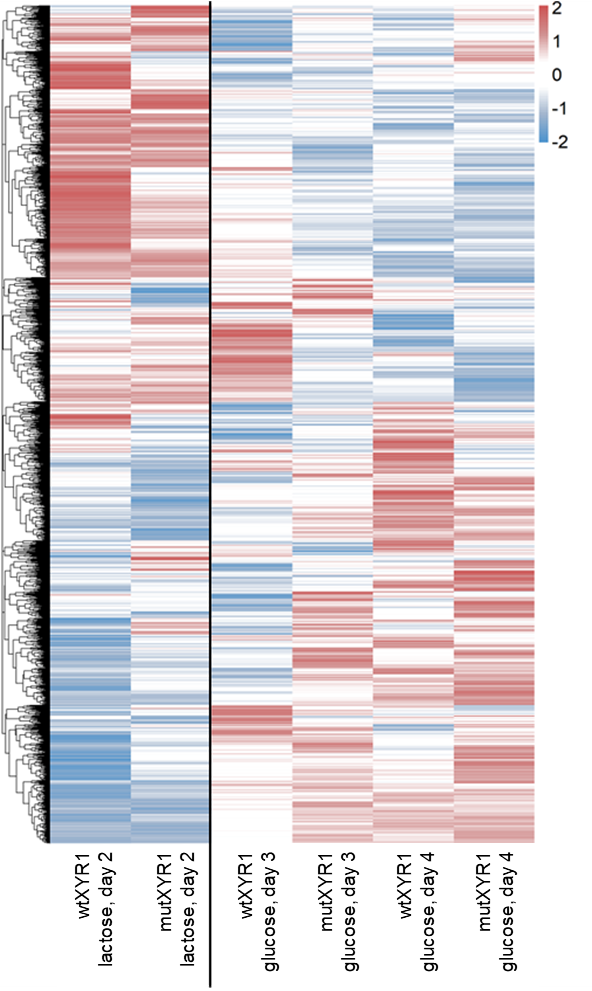


Figure S2. Expression heatmap of all genes (9238) in T. reesei, depicting the difference from average expression for each gene. Average of variance stabilized transformation values was calculated for all the biological replicates and values were centered and scaled in row direction.

Figure S3. Number of up- and downregulated genes in the mutXYR1 strain in each timepoint. Log_2_ fold change threshold was set at 1 and adjusted p-value < 0.05 was used.


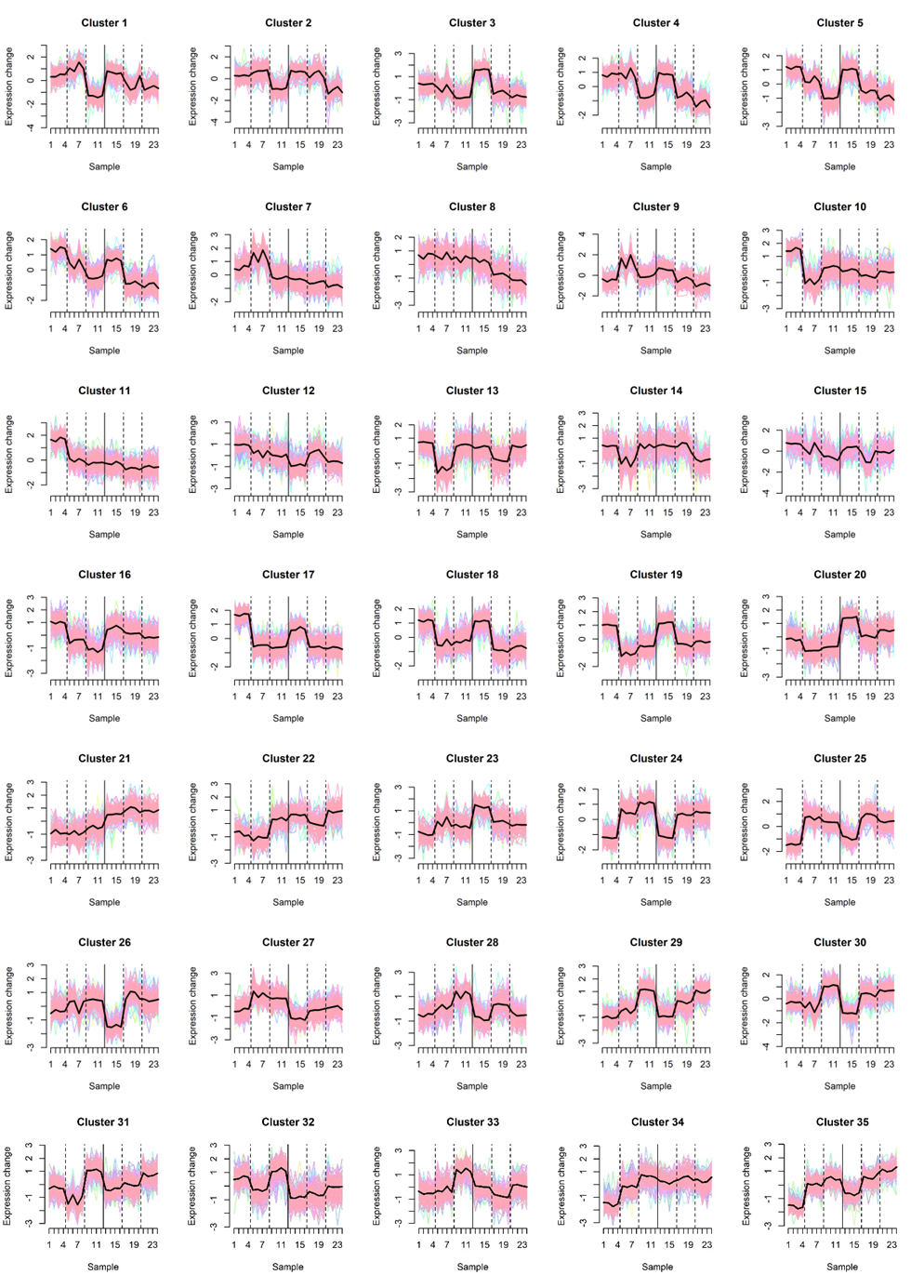


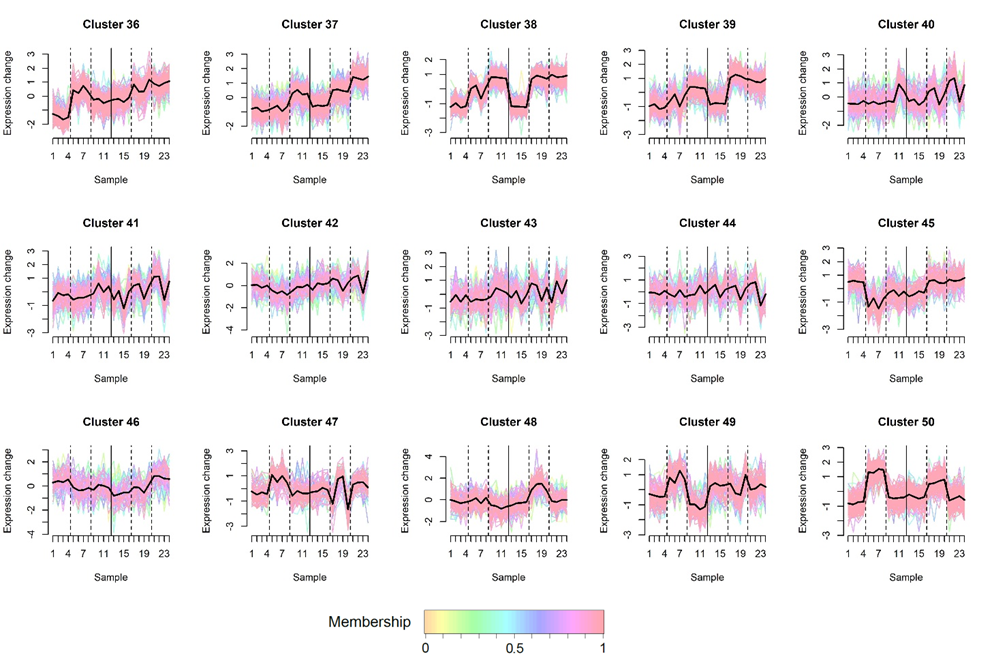


Figure S4. Cluster profiles of the 50 clusters formed when all T. reesei genes were clustered based on their expression change. Samples 1-12 correspond to wtXYR1 strain and samples 13-24 correspond to mutXYR1 strain. Biological replicates are shown separately, and the dotted vertical lines separate the timepoints from each other. The horizontal black line in the middle is the cluster center and the colored lines represent the expression profiles of individual genes. The color of the line indicates the membership value. The higher the membership value, the better the gene fits in the cluster.


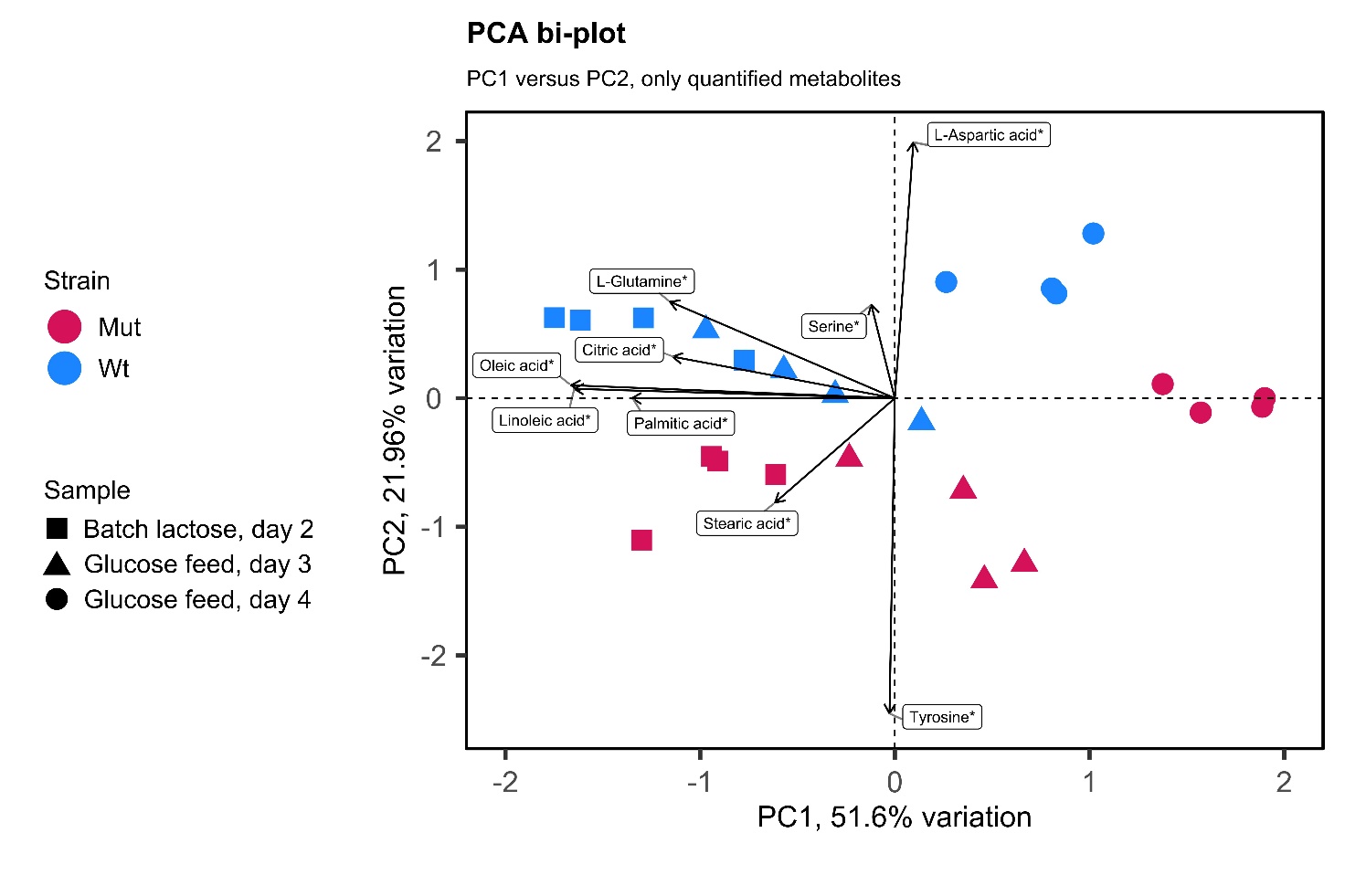


Figure S5. PCA plot of all quantified metabolites from all three time points together. Different timepoints are represented by the different colors and wtXYR1 samples are marked with circles and mutXYR1 samples are marked with triangles. Arrows represent metabolite loadings, i.e., the direction and weight of how the metabolites affect the principal components.


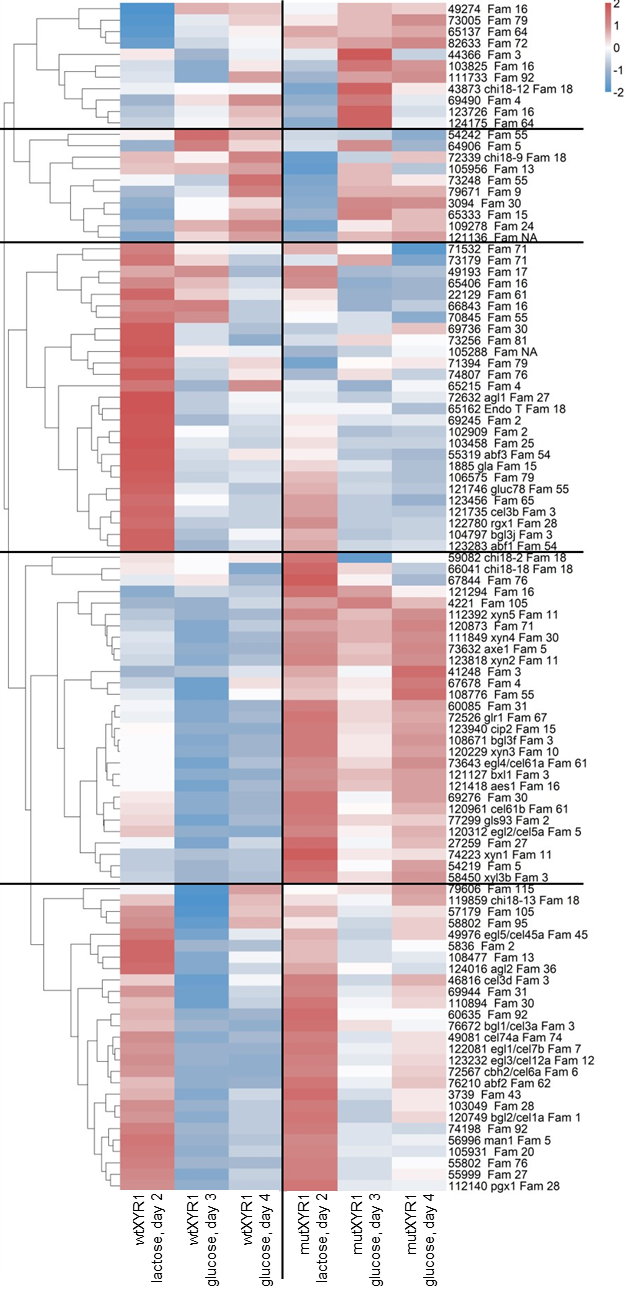


Figure S6. Heatmap of differentially expressed CAZys genes (log_2_ fold change > 1 or < -1, adjusted p-value < 0.05 in at least one timepoint) according to Häkkinen et al. [34]. Average of variance stabilized transformation values was calculated for all the biological replicates and values were centered and scaled in row direction. The vertical black line separates the wtXYR1 and mutXYR1 samples from each other and the horizontal black lines separate different clusters of differentially expressed CAZy genes. Gene IDs, common names and CAZy family numbers are shown on the right.
